# Histologic atlas of the Egyptian rousette (*Rousettus aegyptiacus*) with an integrated review of macroscopic anatomy

**DOI:** 10.1186/s13567-026-01787-x

**Published:** 2026-08-27

**Authors:** Jessica A. Elbert, Brian R. Amman, Tara K. Sealy, Patrick Atimnedi, Jonathan S. Towner, Elizabeth W. Howerth

**Affiliations:** 1 Department of Pathology, University of Georgia College of Veterinary Medicine, Athens, GA USA; 2 Viral Special Pathogens Branch, Division of High-Consequence Pathogens and Pathology, Centers for Disease Control and Prevention, Atlanta, GA USA; 3 Uganda Wildlife Authority, Kampala, Uganda

**Keywords:** Egyptian rousette, bat, *Rousettus aegyptiacus*, histology, pathology, natural reservoir

## Abstract

The Egyptian rousette (*Rousettus aegyptiacus*) has garnered escalating scientific interest owing to its remarkable physiological adaptations, its role as a natural reservoir for emerging zoonotic pathogens, and its potential as a model organism for comparative mammalian investigations. Despite this interest, comprehensive descriptions of normal tissue architecture in this species remain limited and fragmented, hindering interpretation of experimental and spontaneous disease processes. Here, we present a comprehensive histologic atlas derived from wild-caught Egyptian rousette specimens, documenting normal tissue architecture across major organ systems and establishing a standardized reference for future investigations. To contextualize these findings, we also integrate previously published descriptions of Egyptian rousette anatomy and histology, providing a consolidated summary of existing knowledge. This atlas serves as a foundational reference for studies of physiology, immunobiology, and pathology in Egyptian rousettes, facilitating comparative analyses, supporting investigations of host-pathogen interactions, and advancing broader understanding of chiropteran biology relevant to biomedical research, wildlife health, and public health surveillance.

## Introduction

Bats, belonging to the order Chiroptera (etymologically derived from the Greek "cheir" meaning hand and "pteron" meaning wing), represent one of the most taxonomically diverse mammalian orders. With 1500 recognized species as of August 2025, chiropterans constitute approximately 20% of all mammalian biodiversity, ranking second only to rodents in species richness [1]. As the sole mammals capable of sustained, powered flight, bats have successfully colonized diverse ecological niches across all continents except Antarctica, exhibiting extraordinary ecological, anatomical, and physiological diversity that is virtually unparalleled within the animal kingdom [1, 2]. Paleontological evidence indicates that bats have existed for at least 52 million years, with early fossil specimens displaying fully developed wing structures, suggesting that powered flight evolved early in their evolutionary lineage [3]. Contemporary phylogenetic analyses indicate that all extant bat species descended from a common volant ancestor that exhibited many characteristics recognizable in modern chiropterans, including echolocation capabilities and dietary specializations for frugivory and nectarivory. The order Chiroptera is divided into two major suborders: Yinpterochiroptera, encompassing pteropodid fruit bats and several microchiropteran families, and Yangochiroptera, comprising the remaining microchiropteran lineages. These taxonomic groups demonstrate marked differences in echolocation strategies, dietary preferences, and immune gene evolution, reflecting deep phylogenetic divergence within the order [4, 5].

The Egyptian rousette (ERB; *Rousettus aegyptiacus*) is a Yinpterochiropteran bat in the family Pteropodidae with a broad geographic distribution spanning much of Africa, western Asia, the Mediterranean basin, and the Indian subcontinent. Adults are medium-sized fruit bats, weighing 80-180 g (mean approximately 130 g), with body lengths averaging 15 cm and wingspans of approximately 60 cm [6–8]. Males are typically slightly larger than females. ERBs have a canine-like head with a broad muzzle, large eyes, and rounded ears, and the body tapers from broad shoulders to a narrower abdomen. A short, externally visible tail measuring approximately 10 mm is present [7, 9, 10]. Morphologic and genetic analyses recognize six subspecies that differ in geographic range, body size, and pelage coloration: *R. a. unicolor* Gray, 1870; *R. a. aegyptiacus* Andersen, 1912; *R. a. arabicus* Andersen, 1912; *R. a. leachii* Andersen, 1912; *R. a. princeps* Juste and Ibanez, 1993; and *R. a. tomensis* Juste and Ibanez, 1993 [6, 7].

**Figure 1:**
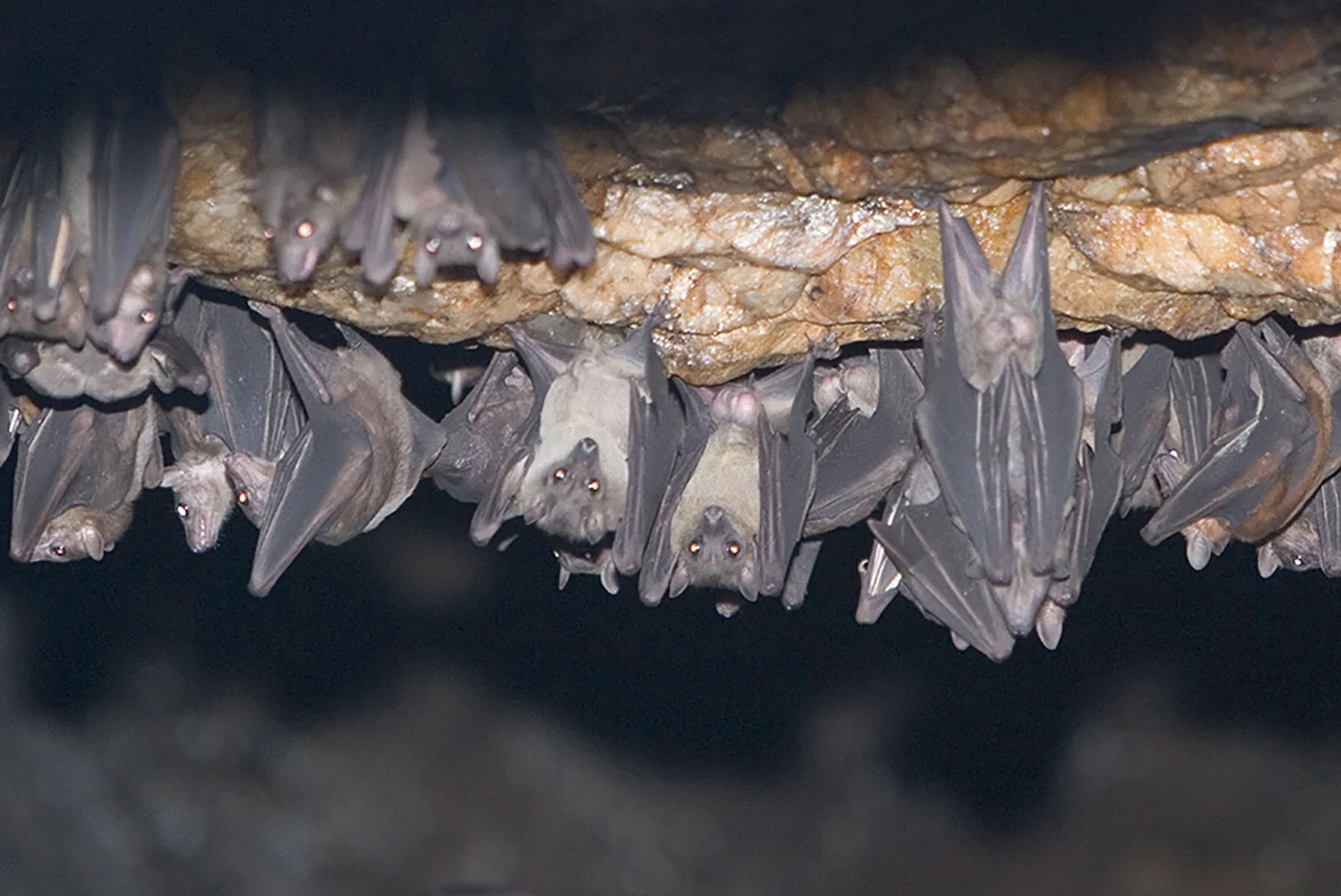
**Egyptian rousette bats (*Rousettus aegyptiacus*) roosting in Kitaka Mine, Uganda.** Shown are a pregnant female (left, forward-facing) and an adult scrotal male (right, forward-facing). Image courtesy of Chris Black.

Egyptian rousettes are predominantly cave-dwelling, highly gregarious animals that form densely aggregated roosts containing up to 100,000 individuals (Figure 1) [6]. While ERBs have been documented in arid biomes, they demonstrate preference for tropical rainforest and deciduous forest environments characterized by abundant canopy cover, suitable roosting opportunities, and diverse fruiting trees [6]. They are frugivorous, preferentially consuming soft, pulpy fruits, including figs, mangoes, and dates, thereby fulfilling critical ecological roles as pollinators and seed dispersers [10]. In tropical regions, ERBs exhibit biannual reproductive cycles with gestation periods of approximately 106 days [6]. Females typically produce singleton offspring that are carried by the dam for 6-8 weeks until the pup achieves independent roosting capability [10]. Juvenile independence occurs between 3 and 6 months of age, with sexual maturity attained between 14 and 18 months [10]. Free-ranging ERBs are estimated to live on average up to 2 years [11]; however, anecdotal observations suggest free-ranging ERBs may live much longer. The maximum recorded lifespan of individuals in managed care is 25 years [6]. ERBs represent one of the few megachiropteran species capable of echolocation, producing characteristic short, repetitive tongue clicks against the oral cavity to facilitate navigation [10, 12–14,15]. Within Uganda, natural predation pressure includes large raptorial species such as the Lanner falcon (*Falco biarmicus*), Palm-nut vulture (*Gypohierax angolensis*), and African fish eagle (*Icthyophaga vocifer*); mammalian carnivores such as Genets (*Genetta* spp.); and cave-dwelling reptiles, including cobras (*Naja* spp.), pythons (*Python* spp.), and Nile monitors (*Varanus niloticus*) (J. Towner, personal communication).

ERBs assume epidemiological significance as natural reservoir hosts for both Marburg virus (MARV) and Ravn virus (RAVV), serving as subjects in numerous longitudinal ecological investigations [16–21]. MARV and RAVV, phylogenetically related to Ebola virus, constitute the only recognized members of the species *Orthomarburgvirus marburgense* (family *Filoviridae*, genus *Orthomarburgvirus*) and serve as the etiological agents of Marburg virus disease (MVD) in humans-a severe viral hemorrhagic fever typically emerging in sub-Saharan Africa, characterized by human-to-human transmission and case fatality ratios reaching 90% [22, 23]. In humans, Marburg virus disease typically begins with an acute febrile illness marked by fever, headache, fatigue, myalgia, and prominent gastrointestinal signs such as vomiting, diarrhea, and abdominal pain, with hemorrhagic manifestations (e.g., hematemesis and bloody diarrhea) developing in a subset of patients during later stages of disease [24]. Fatal outcomes are driven by viral tropism for monocytes, macrophages, hepatocytes, and endothelial cells, leading to dysregulated inflammatory responses, vascular leakage, coagulopathy, and progressive hypovolemic shock with multiorgan failure [25]. In contrast to the severe, frequently fatal disease observed in humans infected with MARV and RAVV, ERBs remain clinically healthy following experimental inoculation, developing low-level viremia with widespread, multi-organ viral dissemination detectable through molecular diagnostics [18, 26]. Histologic lesions of MARV-infected ERBs are characterized by mild, transient hepatitis with small, randomly scattered aggregates of macrophages and lymphocytes, occasional neutrophils, necrotic or apoptotic hepatocytes, karyorrhectic debris, and minimal hemorrhage [18].

The ERB has garnered increasing scientific interest in recent years due to its relevance at the intersection of public health and ecological research, its ability to maintain good health and reproductive performance under managed care, and its distinctive features of mammalian biology and immunology, making it a valuable model organism for comparative mammalian studies. Despite this growing research attention, comprehensive characterizations of the species as a complete biological entity, extending beyond their role as a viral reservoir host, remain notably limited and fragmented across the scientific literature. The objective of this study was to characterize the normal histologic architecture of major organ systems in Egyptian rousette bats using tissues derived from clinically healthy, wild-caught individuals. By integrating original histologic evaluation with existing chiropteran literature, this atlas provides a reference framework for interpreting tissue morphology in this important species. These data are intended to support future investigations requiring accurate baseline histology, including comparative anatomy, experimental pathology, and studies of host-pathogen interactions, while also contributing to a broader understanding of chiropteran biology relevant to public health surveillance and conservation.

## Materials and methods

### Study population and sample collection

A total of 69 free-ranging Egyptian rousettes [juvenile = 49; adult = 20 (forearm length > 89 mm [17, 27]); male = 35; female = 34], were euthanized and collected during 2008-2009 as components of investigative efforts conducted by the Centers for Disease Control and Prevention (CDC) to identify the orthomarburgvirus natural reservoir host [16, 17]. All capture, processing, and experimental procedures were performed in strict accordance with institutionally approved animal care and use protocols, as previously detailed [16, 17]. Specimen collections were completed under Uganda Wildlife Authority approval and conducted following American Veterinary Medical Association guidelines for euthanasia and National Research Council recommendations for laboratory animal care and use [17]. Bats were individually euthanized via an overdose of isoflurane, followed by cardiac exsanguination. At the time of capture, whole blood and splenic tissues were collected for Marburg virus quantitative reverse transcription polymerase chain reaction (RT-qPCR) analysis (negative = 41; positive = 27; unknown = 1). RT-qPCR procedures were performed as previously described [16]. Mild to moderate, multifocal, lymphoplasmacytic hepatitis was identified in a subset of Marburg-virus-positive ERBs and closely resembled the histopathologic lesions described in experimentally Marburg-virus-infected ERBs [18, 28]. No additional tissue morphologic changes attributable to viral infection were identified.

### Specimen preparation and fixation

At the time of initial field collection, abdominal and/or thoracic cavities were opened to facilitate penetration of 10% neutral buffered formalin, with carcasses fixed as intact specimens. Following initial formalin fixation, specimens were transferred to 70% ethanol for long-term preservation; definitive time points for this transfer are not documented. Specimens were subsequently maintained in fixative in temperature-controlled storage within plastic gallon pails with screw-top lids for approximately 15 years. To the authors' knowledge, the specimens were never frozen.

### Histologic processing and analysis

In 2023, comprehensive necropsies were performed on each formalin-fixed specimen by a single pathologist (J.A.E.). All work with Egyptian rousette specimens was conducted under Biosafety Level 2 (BSL-2) conditions in accordance with CDC biosafety policies and the guidelines outlined in the Biosafety in Microbiological and Biomedical Laboratories (BMBL) [29]. As 10% neutral buffered formalin has been proven to effectively inactivate high-consequence pathogens such as Ebola virus, Nipah virus, and Lassa virus, no additional biosecurity measures were deemed necessary [30]. Necropsy and tissue handling were performed in an American Association for Accreditation of Laboratory Animal Care (AAALAC)-accredited animal facility under the oversight of the CDC Institutional Animal Care and Use Committee (IACUC), following institutional safe handling, decontamination, storage, and carcass disposal protocols.

Representative tissue sections were collected from all specimens, including cardiac, tracheal, pulmonary, lymphoid, hepatic, renal, adrenal, lingual, gingival, esophageal, gastric, pancreatic, intestinal, cutaneous, patagial, salivary, adipose, thymic, thyroidal, otic, ocular, nasal turbinate, neural, muscular, and reproductive tissues. The following tissue-collection protocol was used: a full longitudinal section of the heart, including the great vessels and thymus; a proximal cross-section of the esophagus and trachea at the level of the thyroid glands; cross-sections of each lobe of multilobed organs (lung, liver, and salivary glands), lymph nodes, and reproductive tissues; longitudinal sections of the kidney including the adrenal gland; multiple cross-sections of all gastrointestinal segments; serial coronal sections of the entire head including the mandible; a horizontal section of the left pectoral muscle with overlying dermal tissue; a central section of plagiopatagium; and longitudinal sections of the pelvis including spinal cord.

Histologic processing was performed using a HistoCore PEGASUS tissue processor (Leica Biosystems, USA). Tissues were embedded using a Tissue-Tek TEC embedding station (Sakura Finetek, USA), sectioned with an RM2255 microtome (Leica Biosystems, USA), floated on a Healthcare Tissue Flotation Bath (Fisher Scientific, USA), and stained using a Tissue-Tek Prisma Plus automated stainer (Sakura Finetek, USA). Tissue specimens were fixed, trimmed, and held in ambient formalin for 1-3 h prior to processing, including tissues previously fixed in ethanol. Specimens were decalcified in Kristensen's decalcifying solution as needed. The initial processing step consisted of formalin for 44 min, followed by four sequential alcohol changes of 30 min each, one alcohol change of 60 min, and one alcohol change of 90 min. This was followed by two xylene changes of 45 min each and one xylene change of 90 min. Paraffin infiltration consisted of two paraffin changes of 60 min each and a final paraffin change of 80 min, during which tissues remained until embedding, typically for an additional 1-2 h. Alcohol, xylene, and paraffin concentrations increased progressively according to the Pegasus processor concentration algorithm. Tissues were processed using standard histologic techniques and embedded in liquid paraffin (Paraplast Plus, Leica Biosystems, USA) using standard embedding methods. Serial sections were cut at 4 μm thickness, mounted on glass slides, and stained with hematoxylin and eosin (H&E) using conventional protocols. All histologic procedures were performed in accordance with institutional standard operating procedures and complied with American Association of Veterinary Laboratory Diagnosticians (AAVLD) accreditation standards. Positive control slides were included for all staining procedures, including H&E, special stains, and immunohistochemistry, to ensure staining quality.

All histologic sections generated from each specimen were examined. Each organ from each individual was represented on at least one slide, with larger organs typically represented on two to three slides. In total, tissues from each individual were distributed across 5-8 slides, resulting in evaluation of more than 5000 tissue sections overall. Representative images were selected following review of the complete dataset and were chosen on the basis of tissue preservation, staining quality, minimal processing artifact, and clear demonstration of typical histologic architecture for the organ of interest. Images were selected to illustrate features consistently observed across the cohort.

Given the anatomical complexity of Egyptian rousette bats and the need to maintain a focused and representative histologic survey, this atlas does not include every tissue or organ system. Instead, emphasis was placed on larger, functionally significant organs that provide broad characterization of species histology and serve as a reference framework for future comparative and pathological investigations.

### Use of tissues from managed care ERBs

Due to the prioritization of splenic tissue from wild-caught specimens for Marburg virus RT-qPCR analysis at the time of capture, splenic histologic images presented herein were obtained from 5 to 7-month-old, managed care ERBs maintained within the CDC research colony, providing representative splenic histoarchitecture for atlas completion. Similarly, animals used for in situ and ex situ imaging of male and female reproductive anatomy were sourced from the same colony, as all wild-caught specimens had been previously processed for histologic analyses.

## Literature review and histologic atlas

### Integument

**Figure 2:**
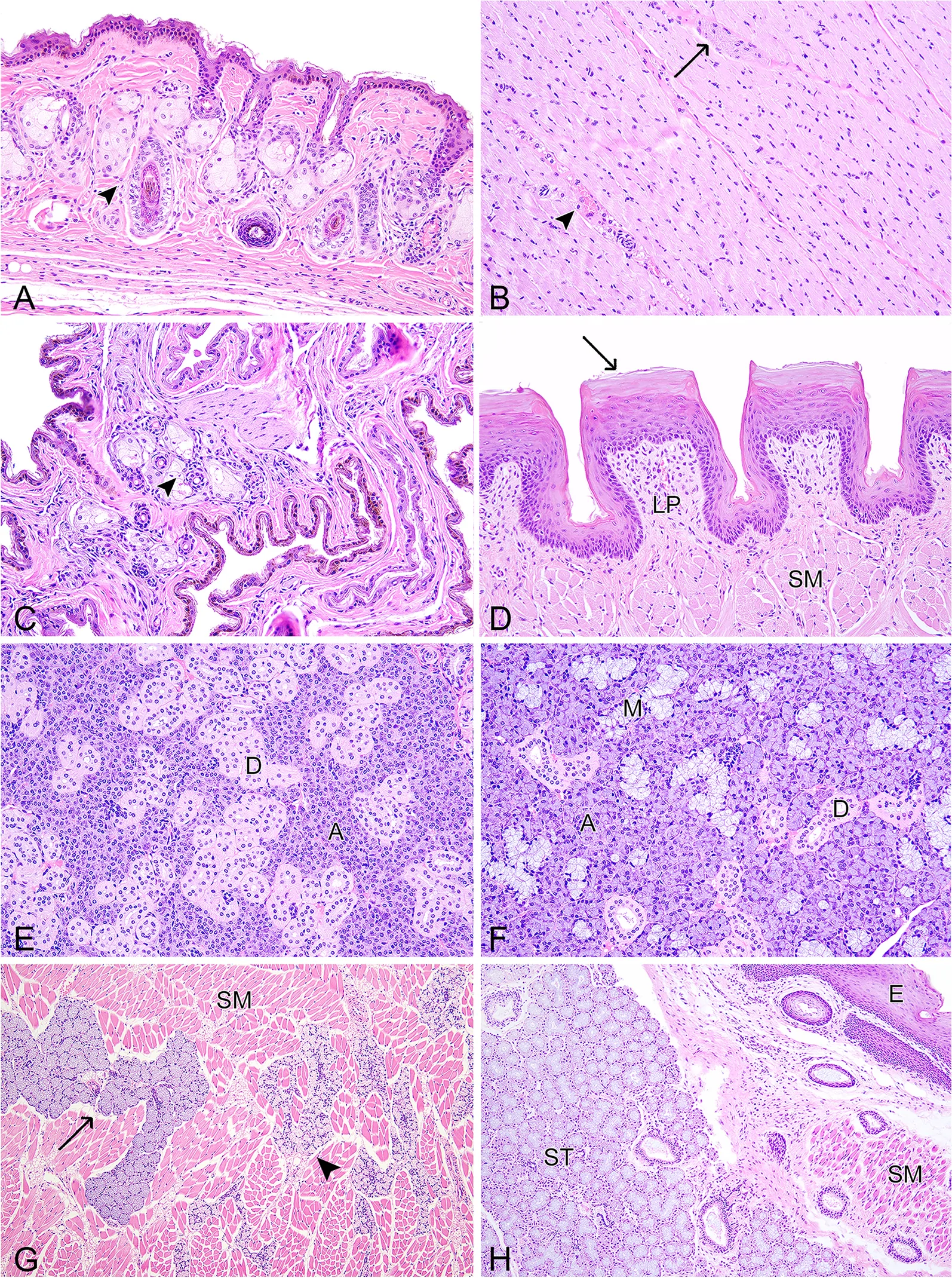
**Histologic features of haired skin, skeletal muscle, wing membrane, tongue, and salivary tissue in Egyptian rousettes (*Rousettus aegyptiacus***). ** A** Haired skin from the dorsal head with multiple pilosebaceous units (arrowhead). 20, hematoxylin and eosin (H&E). **B** Longitudinal section of pectoral skeletal muscle with small caliber vessel (arrowhead) and nerve (arrow) in a juvenile female. ×20, H&E. **C** Wing membrane (plagiopatagium) with pilosebaceous units (arrowhead) in a juvenile male. ×10, H&E. **D** Tongue in a juvenile male with filiform papillae containing keratinizing stratified squamous epithelium (arrow) overlying the lamina propria (LP) and subjacent skeletal muscle (SM). ×20, H&E. **E** Parotid salivary gland with serous acini (A) and striated ducts (D) in an adult female. ×20, H&E. **F** Submandibular salivary gland with serous acini (A), striated ducts (D), and mucous acini (M) in an adult female. ×20, H&E. **G** Serous secreting von Ebner's glands (arrowhead) and mucous secreting Weber's glands (arrow) intercalating between lingual skeletal muscle (SM) in a juvenile male. ×10, H&E. **H** Serous salivary tissue (ST) in the soft palate of an adult male with keratinizing stratified squamous epithelium (E). ×4, H&E. SM, skeletal muscle.

The ERB pelage is short (approximately 5 mm), ranging from dark brown to medium gray with paler ventral coloration, and uniformly distributed across dorsal and ventral surfaces [7]. Facial fur is longest on the forehead and shorter elsewhere, covering nearly the entire face to the tip of the muzzle [7]. Fur extends onto the proximal forearm on both surfaces, reaching the ankles dorsally but only to the elbow ventrally [7]. Frequently, there is a distinctive pericervical collar of pale yellow or orange fur and two specialized glandular regions of stiffer hairs on the lateral cervical aspects [6, 9, 10]. The integumentary characteristics underlying the pericervical collar are distinguished by thin epidermal layers (1-2 cell thickness) with subjacent dermal nests of hair-follicle-associated alveolar sebaceous glands that are morphologically similar to, but larger than, those in other body regions (Figure 2A) [31]. These glands are twice as large in males compared with females, and are hypothesized to function in individual recognition and chemical communication [31]. In the haired skin sections examined in the present study, including the dorsolateral cranial dermis, dermis overlying the pectoral musculature, dermis overlying the pelvis, and wing membrane, apocrine glands were identified only within the haired skin overlying the lateral aspects of the face.

### Skeletal system

The ERB skeletal architecture has been comprehensively characterized [6, 9, 10, 32, 33,34]. The vertebral column comprises 7 cervical, 13 thoracic, 5 lumbar, 6 fused sacral, and 5 caudal vertebrae. The sternum features three prominent keels providing enlarged attachment surfaces for the pectoralis musculature. The thoracic cage consists of 13 ribs (7 sternal, 6 vertebral) and the pectoral girdle includes elongated, narrow scapulae.

Numerous skeletal adaptations facilitate powered flight, including increased digit diameters in planes experiencing maximal bending forces and relatively shortened metacarpals enabling high wing camber during downstroke while reducing drag during upstroke [33, 35]. The powerful musculus palmaris longus additionally contributes to the high camber of the wing. ERBs possess highly specialized pectoralis muscles (pars posterior) optimized for flight power generation [36]. The elbow flexion/extension muscles (biceps brachii and triceps brachii) demonstrate comparable physiological cross-sectional areas but shorter fiber lengths relative to the pectoralis, indicating optimization for large force generation [36]. ERBs have enlarged pectoralis and elbow muscles resembling avian rather than non-volant mammalian configurations (Figure 2B) [36]. Type IIa and Type IIa/x muscle fibers constitute the predominant muscle mass components, with infrequent Type I and Type IIx fiber detection [37]. Notably, ERB capillary density in skeletal muscle, representing oxygen and nutrient delivery efficiency, was lower than in insectivorous species *Myotis myotis* and *Molossus ater*, potentially reflecting flight pattern variations [38].

### Wing membranes

*Rousettus leschenaultii* bats, a closely related species to ERBs, become volant at approximately 6 weeks of age, a time frame that also aligns with anecdotal observations in ERBs (J. Towner, personal communication) [39]. ERB wing membrane architecture follows typical chiropteran organization, comprising the uropatagium (tail membrane); plagiopatagium extending from the dorsum to the fifth digit; the dactylopatagium (chiropatagium) extending between phalanges and subdivided into major, medius, minus, and brevis components; and the propatagium extending from shoulder to thumb, forming the wing's leading edge [40, 41].

Tissue composition varies among the plagiopatagium, dactylopatagium, and uropatagium, with general architecture consisting of stratified squamous epithelial bilayers (ventral and dorsal) separated by connective tissue containing skeletal muscle, pilosebaceous units, vascular and lymphatic networks, collagen, and linearly arranged elastin fibers (Figure 2C) [40, 41]. Strongly tapered, tactile hairs on dorsal and ventral wing surfaces facilitate airflow sensing for enhanced flight maneuverability [42].

ERBs have claws on the terminal phalanges of the first and second thoracic limb digits, with the first digit being opposable [6, 41]. Compared with avian species, ERBs demonstrate reduced wingspan variation during flight due to the need to prevent manus folding, which would cause plagiopatagium collapse [43]. Although an early report suggested that ERBs lack a calcar, the bony or cartilaginous structure associated with the first tarsal row that supports and spreads the uropatagium [44], both the present study and more recent literature identify a cartilaginous structure consistent with that described in ERBs [45] and other *Rousettus* species [46, 47].

## Digestive system

### Tongue

The ERB tongue features a concave midline with prominent, abundant mechanical papillae. Seven filiform papillae subtypes vary in shape, size, density, and orientation; fringing degree indicates high frugivorous dietary adaptation [48]. Additional structures include scattered gustatory (fungiform), conical, and circumvallate papillae [49–54]. The dorsal epithelium comprises keratinized stratified squamous epithelium characterized by basal, spinous, and outermost highly keratinized layers (Figure 2D) [49]. ERBs possess an entoglossal plate associated with the lingual root that supports the tongue and features a thin compact bone core with central hematopoietic tissue associated with elastic cartilage [49].

### Salivary glands

**Figure 3:**
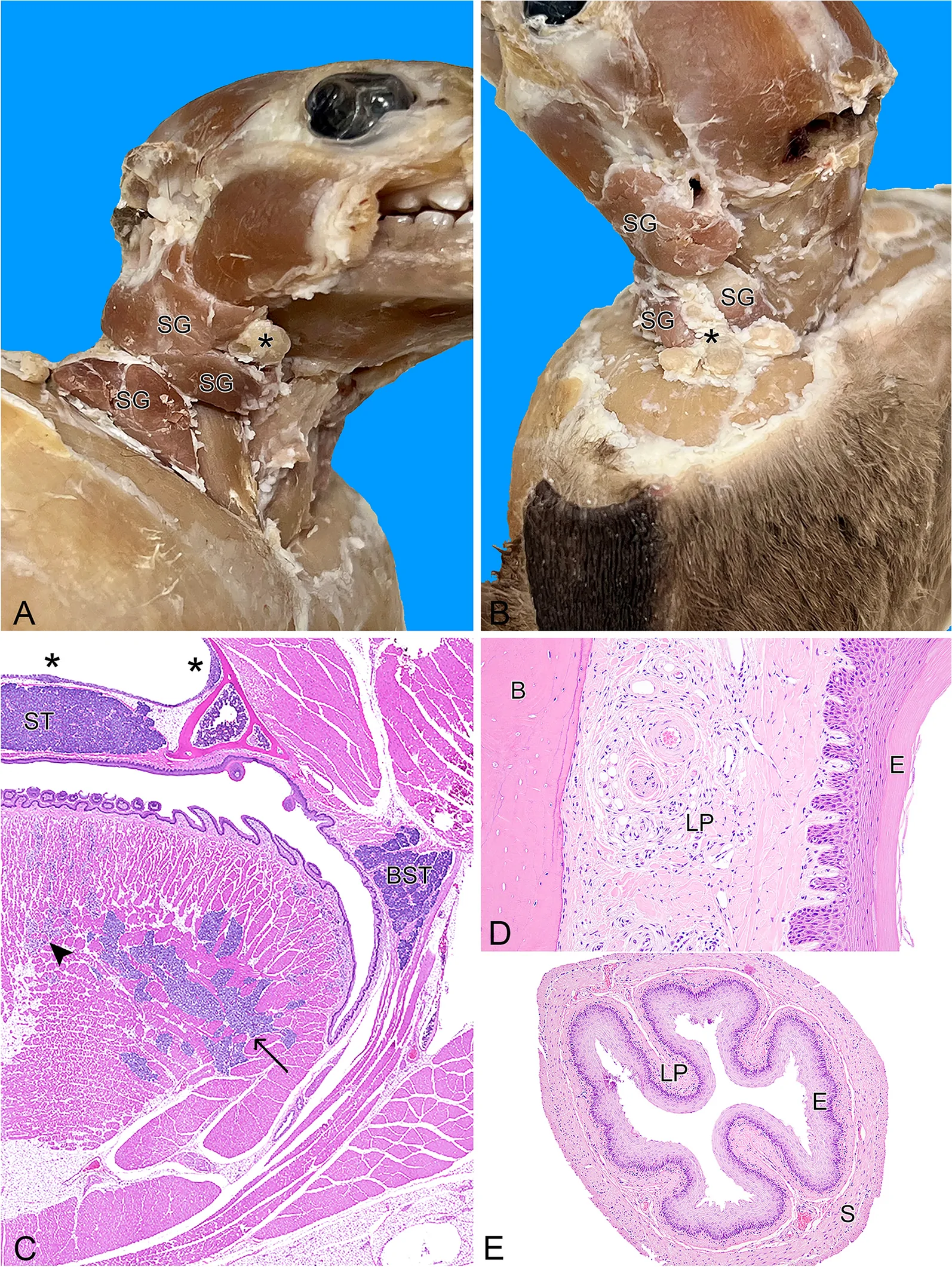
**Gross and histologic features of salivary tissue, oral cavity, gingiva, and esophagus in Egyptian rousettes (*Rousettus aegyptiacus***).** A ** Right ventrolateral view of cervical region showing multi-lobed parotid salivary gland (SG) and submandibular lymph node (asterisk). **B** Left dorsolateral view of the cervical region showing a lymph node chain (asterisk) extending caudally beyond the multi-lobed parotid salivary gland (SG). **C** Cross-section of caudal oral cavity showing tongue with lingual salivary tissue (arrowhead, von Ebner's glands; arrow, Weber's glands), buccal salivary tissue (BST), soft palate salivary tissue (ST), and nasal-associated lymphoid tissue (asterisks) in an adult female. ×0.8, H&E. **D** Gingiva in an adult male with keratinizing stratified squamous epithelium (E) overlying the lamina propria (LP) and bone (B). ×20, H&E. **E** Cross-section of esophagus in an adult male with mucosa composed of non-keratinizing stratified squamous epithelium (E) overlying the lamina propria (LP) and muscularis externa composed of predominantly smooth muscle (S). ×10, H&E.

Salivary gland architecture has been described in *Rousettus amplexicaudatus* bats, another closely related species to ERBs, encompassing principal parotid glands (Figures 2E, 3A, B), accessory parotid glands, accessory submandibular glands, and principal submandibular glands (Figure 2F) [55]. ERBs have lingual salivary glands containing von Ebner's glands (serous-secreting glands expressing acidic mucopolysaccharides) and Weber's glands (mucous-secreting glands producing neutral mucopolysaccharides) intercalating between striated muscles (Figures 2G, 3C) [49], though one publication reports Weber's glands exclusively in median and posterior lingual regions [48]. In frugivorous bats, parotid secretory granules are typically seromucous rather than serous [56]. The current study confirmed hard palate, soft palate (Figure 2H), buccal (Figure 3C), and sublingual mucoid salivary glands, the latter positioned between the rostral mandible and tongue.

### Gastrointestinal tract

ERB gingiva is lined by stratified squamous epithelium supported by a lamina propria closely associated with underlying bone (Figure 3D). The esophageal wall is composed of mucosa lined by non-keratinizing stratified squamous epithelium, supported by the lamina propria and muscularis mucosa, followed by the submucosa and an outer muscularis externa (Figure 3E). The muscularis externa is composed predominantly of striated muscle in the proximal esophagus, which transitions progressively to predominantly smooth muscle in the distal esophagus.

**Figure 4:**
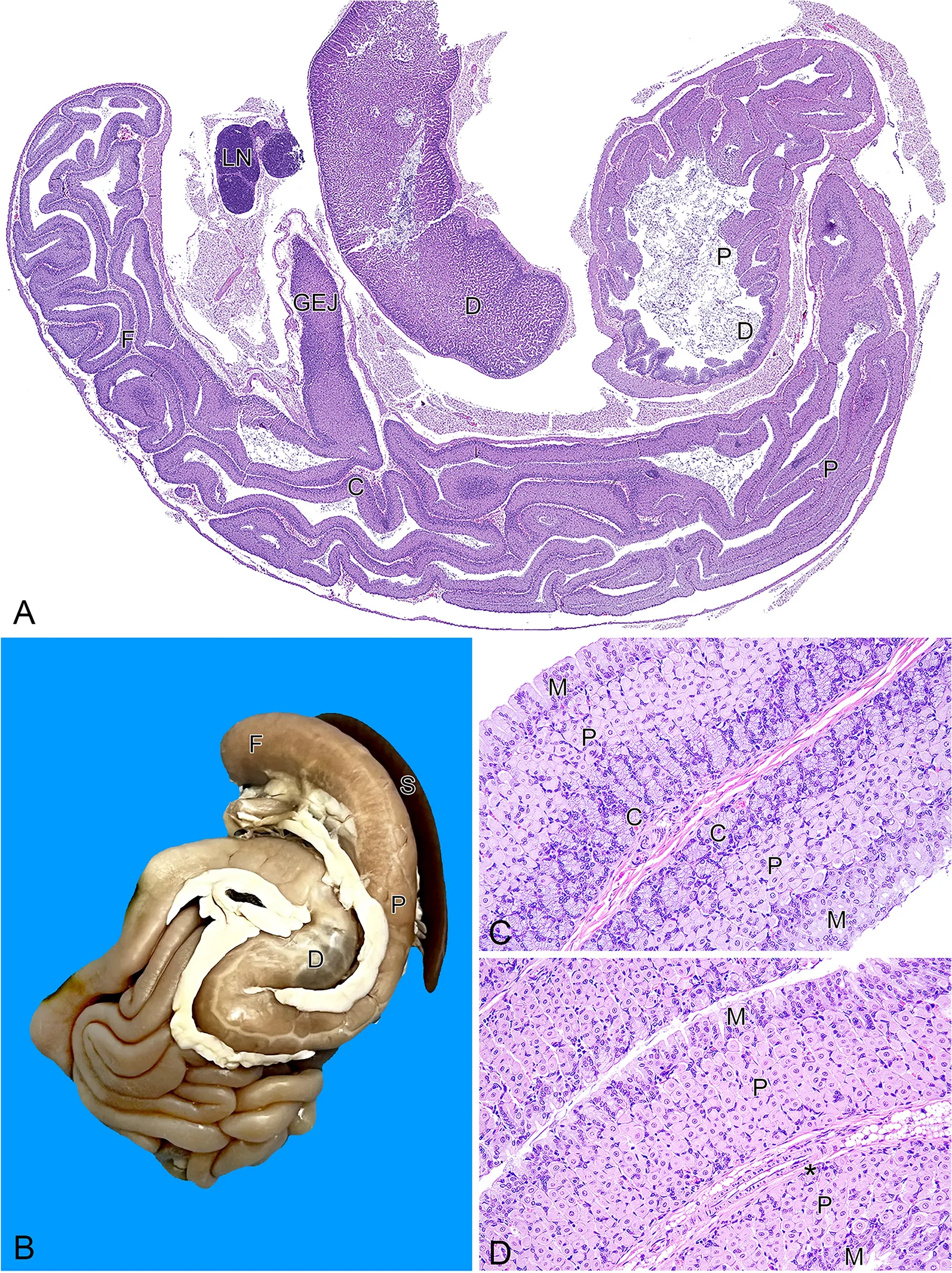
**Gross and histologic features of the stomach in Egyptian rousettes (*Rousettus aegyptiacus***). ** A** Subgross image of the stomach, showing the gastroesophageal junction (GEJ), fundus (F), cardia (C), pylorus (P), and duodenum (D) in an adult female. ×2, H&E. **B** Gross image of the gastrointestinal tract as it appears ex vivo, showing the fundus (F), pylorus (P), and duodenum (D) in an adult female. The spleen (S) is present along the greater curvature of the stomach. **C** Gastric fundus with surface mucous cells (M), parietal cells (P), and chief cells (C) in an adult female. ×20, H&E. **D** Gastric pylorus with surface mucous cells (M), parietal cells (P), and rare chief cells (asterisk) in an adult female. ×20, H&E.

The ERB stomach exhibits an elongated C-shaped morphology, with a long, convex greater curvature and a shorter, nearly straight lesser curvature (Figure 4A, B) [57]. Three anatomic regions include the fundus containing a blunt-ended diverticulum termed the "fundic cecal region" (Figure 4C), the cardia, and the pylorus (Figure 4D) [57]. The pyloric sphincter is externally identifiable by slight diameter constriction and narrow circumferential muscular banding [57]. The stomach comprises four layers: mucosa, submucosa, tunica muscularis, and serosa [48, 57, 58]. Mucosal villi-like folds (rugae) are elongated and curved with fewer gastric glands containing abundant parietal cells, surface and neck mucous cells, and chief cells (Figure 4C, D) [48, 58].

**Figure 5:**
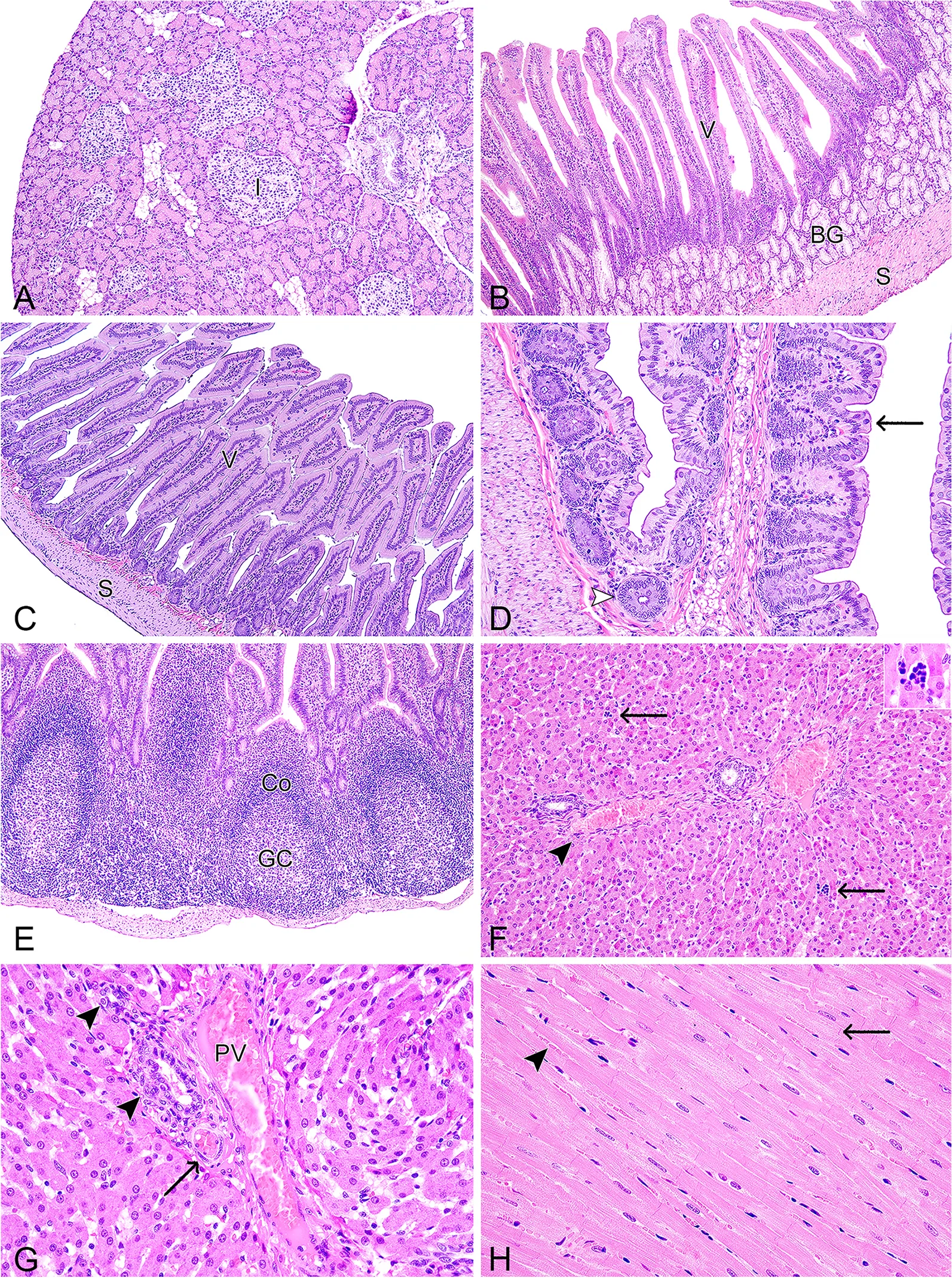
**Histologic features of the pancreas, small and large intestine, gastrointestinal-associated lymphoid tissue, liver, and heart in Egyptian rousettes (*Rousettus aegyptiacus***). ** A** Pancreas showing large endocrine islets (I) in a juvenile male. ×10, H&E. **B** Duodenum with long villi (V) and Brunner's glands (BG) within the submucosa in an adult female. S, smooth muscle. ×10, H&E. **C** Jejunum with long villi (V) in an adult male. S, smooth muscle. ×10, H&E. **D** Colon in an adult female. The mucosa contains numerous crypts of Lieberkühn (arrowhead) and goblet cells (arrow). ×20, H&E. **E** Peyer's patch with numerous germinal centers (GC) and corona/mantle zones (Co) in the ileum of a juvenile male. ×10, H&E. **F** Liver with portal triad (arrowhead) and scant extramedullary hematopoiesis (arrow) in a juvenile male. ×10, H&E. Inset: High magnification of extramedullary hematopoiesis. ×60, H&E. **G** Higher magnification of a portal triad with a portal vein (PV), bile duct (arrowhead), and hepatic artery (arrow). ×40, H&E. **H** Longitudinal section of cardiomyocytes in the right ventricle of an adult female, showing intercalated discs (arrow) and numerous capillaries (arrowhead). ×40, H&E.

ERBs have evolved specialized gastrointestinal tract adaptations enabling efficient and rapid carbohydrate-rich diet digestion, with high carbohydrate paracellular absorption rates compensating for relatively short intestinal tract length [59]. This enables substantial hepatocyte glycogen storage, appearing histologically as feathery cytoplasmic vacuolation diffusely distributed throughout hepatic tissue [41, 59]. The pancreas contributes to glucose absorption, with 9.1% endocrine tissue composition exceeding typical domestic mammalian proportions (Figure 5A) [60]. Endocrine cells are distributed throughout pancreatic islets and as discrete cells within exocrine ducts [60]. Four endocrine cell types have been described: insulin (β) cells (51.4%) distributed throughout islets and extending between glucagon (α) cells (30.6%), with somatostatin (δ) cells (8.8%) and pancreatic polypeptide cells (17.1%) irregularly scattered throughout islets [60].

Small intestinal mucosa contains elongated, anastomosing, apically sharp villi arranged in zigzag patterns containing absorptive enterocytes and goblet cells with elongated, slender microvilli (Figure 5B, C) [48, 61]. Submucosal collagenous fibers are thin and irregular [58]. Occasional Brunner's glands are present within the duodenum (Figure 5B) [62]. Intestinal transit time ranges from 18 to 100 min, with greater than 90% absorption of ingested sugars [63]. Maximum fructose assimilation occurs within 5 min, while maximum glucose uptake occurs within 30 min [64]. Notably, beyond sodium-coupled mediated sugar transport, ERBs rely on passive, paracellular absorption for glucose intestinal absorption to a significantly greater extent than non-volant mammals [65]. Unlike many mammals, the ERB small intestine demonstrates free calcium permeability without active transport mechanisms [6, 66]. The colon is characterized by a short, flat mucosa lacking villi, well-defined crypts of Lieberkühn, and abundant goblet cells (Figure 5D). There is no macroscopic distinction between small intestine and colon. Gastric lymphoid follicles are occasionally observed, while Peyer's patches (Figure 5E) are frequently present within the small intestine. ERBs lack a cecum, appendix, and ascending and transverse colons [62].

## Hepatobiliary system

### Liver and gallbladder

The ERB liver occupies a substantial portion of the cranial abdomen and is anatomically divided into four distinct lobes: a left lobe, medial lobe, a right lobe containing a prominent renal fossa, and a small caudate lobe. The gallbladder is positioned adjacent to the medial and right hepatic lobes on the visceral surface and exhibits an elongated, almost serpentine morphology. Histologically, the liver is characterized by indistinct, roughly hexagonal hepatic lobules centered on centrilobular veins (terminal hepatic venules). Portal tracts, containing hepatic portal veins, hepatic arteries, and bile ducts, are positioned at the vertices of these hexagonal structures (Figure 5F, G). Lymphatic vessels may be observed within portal tracts but frequently collapse during sectioning, rendering them less readily identifiable. Hepatocytes are arranged in densely cellular plates separated by fine vascular sinusoids lined with fenestrated sinusoidal endothelial cells and Kupffer cells, the liver's resident macrophages. In fetal and juvenile specimens, extramedullary hematopoiesis can be observed within the hepatic parenchyma (Figure 5F).

## Cardiovascular system

### Heart

*Rousettus* spp. bats demonstrate larger relative heart size compared with other bat species and exhibit higher vertebral heart scores than mammals generally (Figure 5H) [67, 68]. Great cardiac vessel gross anatomy has been described, with noted venous drainage variations including single left pulmonary vein and two right pulmonary vein groups draining into the left atrium [69]. Additional variations include bilateral cranial vena cava retention as part of upper limb flight requirements [69].

## Nervous system

### Brain

As with other mammals, the brain of pteropid bats is enclosed within a large, bulbous braincase and structurally divided into the forebrain, midbrain, and hindbrain with distinct cerebral hemispheres separated by a longitudinal fissure; the cerebrum consists of a grey-matter cerebral cortex overlying white-matter tracts that connect cortical regions and deeper nuclei, and the hindbrain includes the folded cortex of the cerebellum and brainstem nuclei, reflecting conserved mammalian neuroanatomy of grey matter (neuronal cell bodies) and underlying white-matter fiber pathways [70–72]. The ERB brain, in particular, has been well characterized in the literature [73–77].

**Figure 6:**
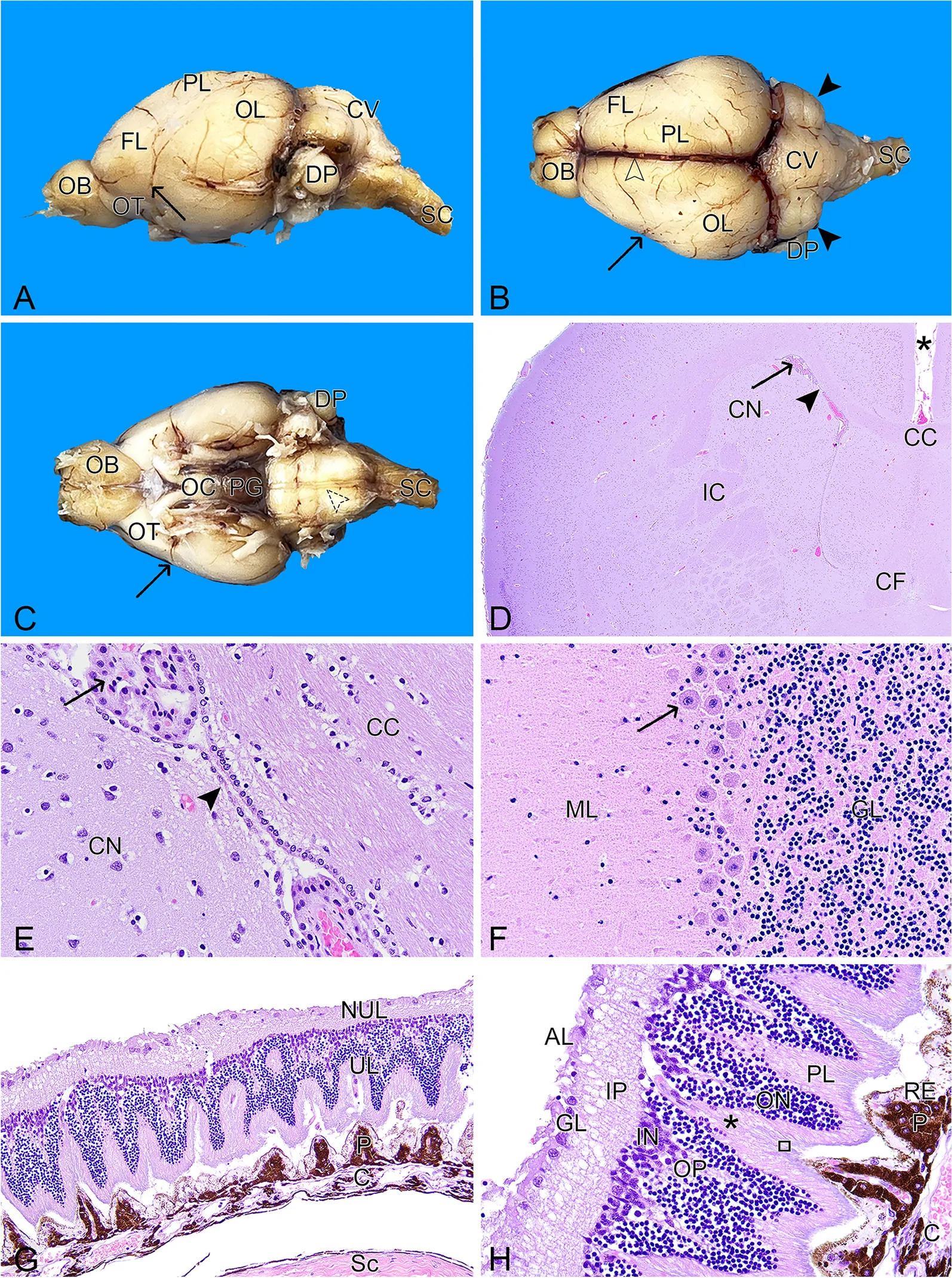
**Gross and histologic features of brain and retina in Egyptian rousettes (*Rousettus aegyptiacus***). ** A** Left lateral, **B** dorsal, and **C** ventral view of the brain of an adult female. The arrow indicates the medial cerebral artery, arrowheads the cerebellar hemispheres, dashed arrowhead the basilar artery, and open arrowhead the medial longitudinal fissure (interhemispheric fissure). DP, dorsal paraflocculus (ventral paraflocculus lost upon removal from calvarium); CV, cerebellar vermis; FL, frontal lobe; OB, olfactory bulb; OC, optic chiasm; OL, occipital lobe; OT, olfactory tubercle; PG, pituitary gland (hypophysis); PL, parietal lobe; SC, spinal cord. **D** Cross-section of the cerebrum at the level of the internal capsule (IC) in an adult female. The arrowhead is pointing to the location of the image provided in panel **E**. ×2, H&E. **E** Higher magnification view of the cerebrum, showing choroid plexus within the lateral ventricle. The arrow indicates the choroid plexus, the arrowhead the lateral ventricle, and the asterisk the medial longitudinal fissure. CC, corpus callosum; CF, column of fornix; CN, caudate nucleus. ×40, H&E. **F** Cross-section of the cerebellum, showing the granular layer (GL), molecular layer (ML), and Purkinje cells (arrow). ×40, H&E. **G** Retina in an adult female. Sclera (Sc), choroid (C) with its conical or spike-like choroidal papillae (P), retina with undulating (UL) and non-undulating (NUL) layers. ×20, H&E. **H** Retina in an adult female. Choroid (C) with its conical or spike-like choroidal papillae (P), retinal epithelium (RE) with granular melanin pigment, photoreceptor layer (PL) comprising the inner segment (square) and outer segment (asterisk), outer nuclear layer (ON), outer plexiform layer (OP), inner nuclear layer (IN), inner plexiform layer (IP), ganglion cell layer (GL), and axon layer (AL). ×40, H&E.

The ERB brain exhibits a characteristic pear-shaped morphology, wider caudally than cranially, with masses ranging from 2.0 to 2.4 g (average: 2.15 g) (Figure 6A-C) [75]. While primordial vomeronasal organ development occurs during early embryogenesis (up to 14 mm crown-rump length), later gestational stages, neonates, and adult *R. leschenaultii* bats lack functional vomeronasal systems [78]. ERBs additionally lack an accessory olfactory bulb, the functional central nervous system relay center for vomeronasal organs [78–81]. Investigations of cholinergic, putative catecholaminergic, and serotonergic neuron distribution and morphology in ERB brains have identified discrete nuclei absent in *Miniopterus schreibersii* bats [73, 82]. Furthermore, hippocampal formation and entorhinal cortex investigations, neural components crucial for learning, memory, and spatial navigation, demonstrate that ERBs possess entorhinal-dentate gyrus projections more similar to primates than rodents [83]. Due to preservation artifacts, interpretation of finer details within the histologic images provided in Figure 6D-F should be approached with caution [84].

ERB spinal cord and brain lipid composition and sphingolipid fatty acids resemble other mammals with differences, particularly in spinal cord composition, theorized to result from gray-to-white-matter ratio variations [85, 86]. Experimental vitamin B_12_ deficiency in managed care ERBs resulted in demyelination of lateral and ventrolateral white matter in caudal cervical and cranial thoracic spinal cord regions, though this has not been documented in free-ranging populations [87].

## Sensory systems

### Eye

The ERB eye exhibits numerous adaptations characteristic of nocturnal animals, including a markedly curved cornea occupying approximately one third of the globe, enlarged anterior and posterior chambers relative to the vitreous, and foveal absence [88]. Extensive studies have characterized ERB retinal structure compared with other chiropteran and mammalian species, particularly nonhuman primates [89–94]. ERB retinas demonstrate retinal decussation patterns identical to other pteropodid bats and primates [74]. As with most pteropodids, ERBs possess avascular retinas with thick retinal layering extending up to or beyond 250 μm [95]. Conical or spike-like choroidal papillae project into the retina, presumably providing nourishment to retinal regions exceeding 140 μm from the vascular choroid (Figure 6G, H) [41, 90, 95]. This undulation increases retinal surface area and photoreceptor density, enhancing nocturnal visual acuity and light-gathering capabilities [88, 90].

Dried eye lens weight has been investigated as an age indicator and may provide more accurate age determination than traditional methods such as forearm length [96]. Harderian glands, lacrimal glands, and well-developed nasolacrimal ducts have been documented in *R. leschenaultii* bats [97].

### Echolocation system

ERBs possess both excellent vision and echolocation capabilities, enabling variable sensory modality use across diverse light conditions and environments [13–15, 98, 99]. ERB echolocation consists of tongue-generated click pairs with 0.6-1 ms duration, frequency ranges of 12-70 kHz, and peak frequencies at 20-40 kHz [13].

The pinnae exhibit high mobility mediated by five individual muscles, with a comparatively large and well-organized population of facial motor nucleus motoneurons supporting sophisticated pinna movements [100]. Echolocation signal timing and pinnae movement investigations have revealed that free-flight echolocation corresponds with downward wingstrokes and forward pinnae movement, with maximal auditory sensitivity occurring when ears are forward-facing [12].

## Respiratory system

### Nasal turbinates

**Figure 7:**
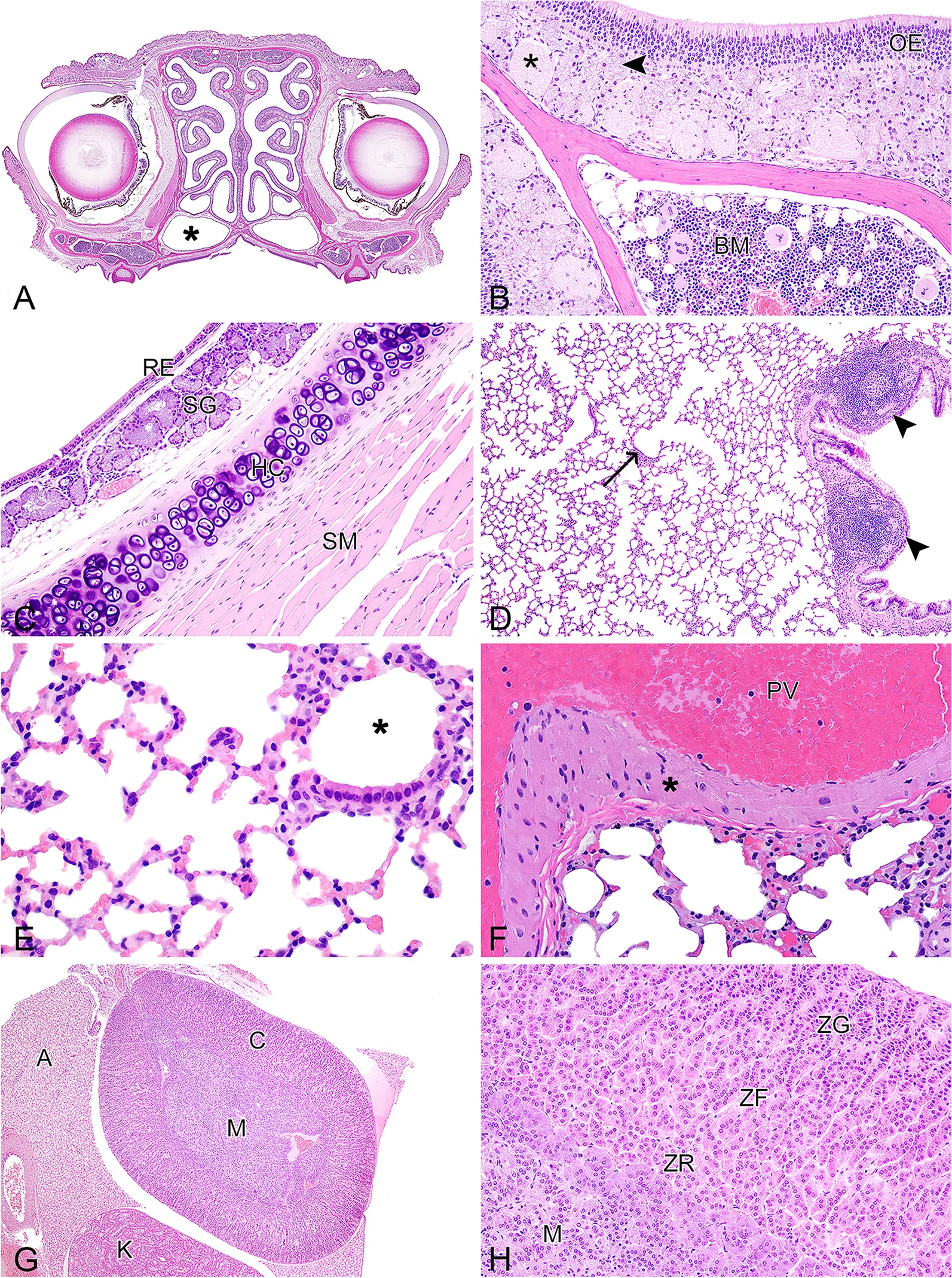
**Histologic features of nasal turbinates, trachea, lung, and adrenal gland in Egyptian rousettes (*Rousettus aegyptiacus***). **A** Coronal cross-section through the nasal turbinates at the level of the eyes. The mandible is not in frame. The asterisk highlights a nasopharyngeal duct. ×0.9, H&E. **B** Nasal turbinates with overlying olfactory epithelium (OE), olfactory nerves (asterisk), olfactory/Bowman's glands (arrowhead), and bone marrow (BM) within nasal turbinate bone in an adult female. ×20, H&E. **C** Trachea with respiratory epithelium (RE), submucosal glands (SG), hyaline cartilage (HC), and striated muscle (SM) in a juvenile male, taken at the level of the thyroid glands. ×20, H&E. **D** Section of lung with a large primary bronchus with bronchus-associated lymphoid tissue (BALT) (arrowhead), terminal/respiratory bronchioles transitioning to alveolar ducts (arrow), and numerous alveoli. ×10, H&E. **E** Terminal/respiratory bronchus (asterisk) transitioning to an alveolar duct. ×60, H&E. **F** Pulmonary vein (PV) with multiple cardiomyocyte layers (asterisk) in a juvenile female. ×40, H&E. **G** Adrenal gland with outer cortex (C) and inner medulla (M), surrounded by adipose (A) and kidney (K) in an adult female. ×4, H&E. **H** Adrenal cortex with zona glomerulosa (ZG), zona fasciculata (ZF), and zona reticularis (ZR) adjacent to the inner basophilic medulla (M) composed primarily of chromaffin cells in an adult female. ×20, H&E.

The nasal turbinate architecture in the ERB exhibits structural characteristics consistent with those previously documented in *R. leschenaultii* bats [101]. This configuration features bilaterally symmetrical frontoturbinal structures and multiple ethmoturbinal complexes that form intricate projections within the nasal capsule, demarcated by a central nasal septum and accompanied by prominent lateral nasopharyngeal ducts (Figure 7A). The nasal turbinates comprise olfactory epithelium overlying olfactory nerves and Bowman's glands, all embedded within vascularized connective tissue and supported by underlying bone (Figure 7B). Notably, the ERB olfactory nerves demonstrate considerable size variation depending on the specific coronal section examined.

### Trachea

The ERB tracheal lumen is lined by respiratory epithelium composed of ciliated pseudostratified columnar epithelium with scattered goblet cells, with submucosal glands present within the deeper lamina propria and submucosa (Figure 7C). The tracheal wall is supported by incomplete rings of hyaline cartilage reinforced by a smooth muscle layer (trachealis muscle).

### Lungs

Investigations across numerous bat species have examined pulmonary morphometric properties and structural adaptations elucidating how bats meet immense oxygen demands required for flight [102–104]. Generally, bats demonstrate overall lung morphology similar to most mammals, with high lung volumes relative to birds or terrestrial mammals, small airspace subdivisions, and extremely thin blood-gas barriers (Figure 7D-F) [102–104]. Bronchus-adjacent hyaline cartilage is limited to primary bronchi and occasionally demonstrates rudimentary ossification [105]. Mucous membrane sections overlying hyaline cartilage are flattened (non-rugated) and connected to subjacent cartilage by connective tissue; bronchial glands and smooth muscle are absent in these regions [105]. Secondary bronchi and beyond are surrounded by well-developed, circumferential smooth muscle layers with prominent bronchial epithelial folding. Bronchiolar epithelium transitions from ciliated pseudostratified epithelium with rare goblet cells in primary bronchi to simple columnar epithelium in secondary bronchi to simple cuboidal epithelium in terminal/respiratory bronchi, before transitioning to thin alveolar epithelium lining alveolar ducts and subsequent alveoli (Figure 7D, E). Similar to rodents, ERB pulmonary veins contain 1-5+ cardiomyocyte layers with larger sarcoplasm than adjacent smooth muscle cells, more readily appreciable in large caliber vessels (Figure 7F) [106].

## Endocrine system

### Adrenal glands

Adrenal glands are organized into a cortex composed of zona glomerulosa, zona fasciculata, and zona reticularis surrounding a central medulla (Figure 7G, H) [107]. ERB adrenal glands are proportionally small relative to body weight and increase during pregnancy [108–110]. A 27% incidence of macroscopic and numerous microscopic accessory adrenocortical bodies has been reported, though these were not observed in the current study [108].

### Thyroid glands

**Figure 8:**
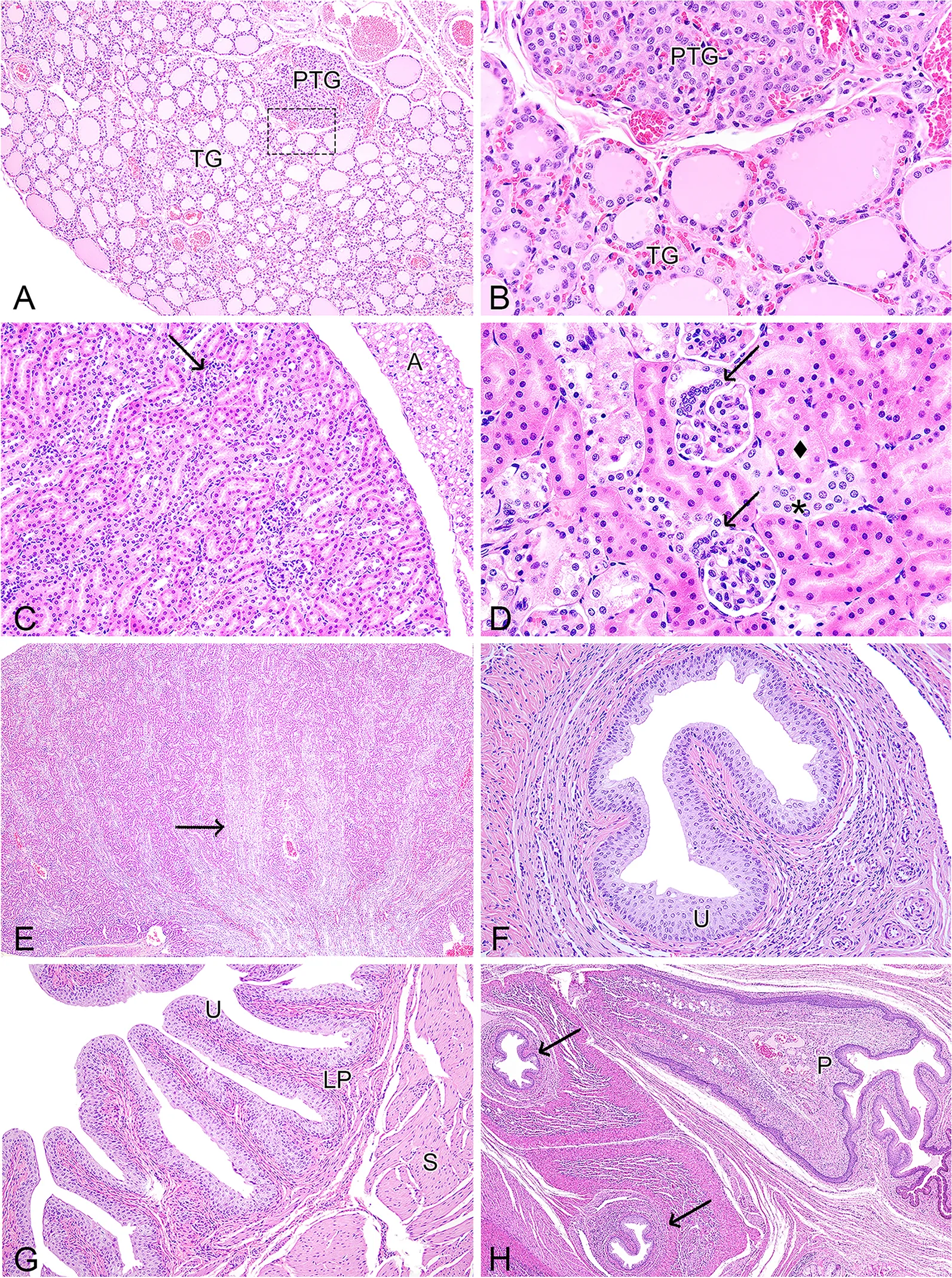
**Histologic features of thyroid gland, parathyroid gland, kidney, urethra, urinary bladder, and penis in Egyptian rousettes (*Rousettus aegyptiacus***). **A** Thyroid gland (TG) with numerous small, colloid-filled follicles with internal parathyroid gland (PTG) in an adult female. ×10, H&E. The dashed rectangle indicates the region shown at higher magnification in panel **B**. **B** Higher-magnification view of the thyroid (TG) and internal parathyroid glands (PTG). ×40, H&E. **C** Renal cortex with fetal glomeruli (arrow) and supracortical brown adipose (A) in a juvenile female. ×20, H&E. **D** Two glomeruli with juxtaglomerular apparatus (arrows), proximal convoluted tubules (diamond), and distal convoluted tubules (asterisk) in the renal cortex of a juvenile female. ×40, H&E. **E** Prominent medullary rays (arrow) in the kidney of a juvenile male. ×4, H&E. **F** Cross-section of urethra lined by urothelium (U) in a juvenile male. ×20, H&E. **G** Urinary bladder with villous-like projections of the lamina propria (LP) lined by urothelium (U) and subjacent smooth muscle (S) in a juvenile male. ×10, H&E. **H** Longitudinal section of retracted penis (P) with cross-sections of penile urethra (arrows) in a juvenile male. ×4, H&E.

ERB thyroid histologic features resemble other mammals, comprising numerous small follicles lined with follicular and parafollicular epithelial cells (Figure 8A, B) [111]. Parathyroid glands composed of chief cell ribbons, oxyphils, and transitional cells were identified in numerous ERBs (Figure 8A, B). While the role of the parathyroid gland has been investigated in hibernating bat species, this organ has not been studied in ERBs to our knowledge [112, 113]. ERBs do not undergo hibernation or torpor [114].

## Urogenital system

### Kidneys

Renal development in ERBs shows fetal glomeruli within the outer cortical nephrogenic zone displaying immature, variably developing morphologies with incompletely formed filtration barriers (Figure 8C), whereas adult glomeruli are uniformly mature and predominantly located in the inner cortex, consistent with expected patterns of prenatal nephrogenesis and postnatal functional maturation [115]. The renal cortex is relatively thick, with few glomeruli in the outermost regions and progressively increasing glomerular density toward the corticomedullary junction, as described in *R. leschenaultii* bats (Figure 8C, D) [116]. Large, numerous medullary rays invade cortical tissue, obscuring defined corticomedullary junctions and conferring a streaming hypercellularity to the tissue (Figure 8E). ERBs possess very short, conical papillae, consistent with reduced urine concentrating ability requirements in frugivorous bats [116]. However, urine specific gravity measurements from free-ranging Egyptian rousettes from Israel indicate substantial urine concentration capabilities [117].

### Urethra and urinary bladder

ERB urethral and urinary bladder histologic features resemble other mammals, comprising mucosa lined by urothelium, a muscularis of smooth muscle, and adventitia (Figure 8F-H). Prominent villous-like projections can be identified in the bladder when not distended (Figure 8G).

### Male reproductive system

**Figure 9:**
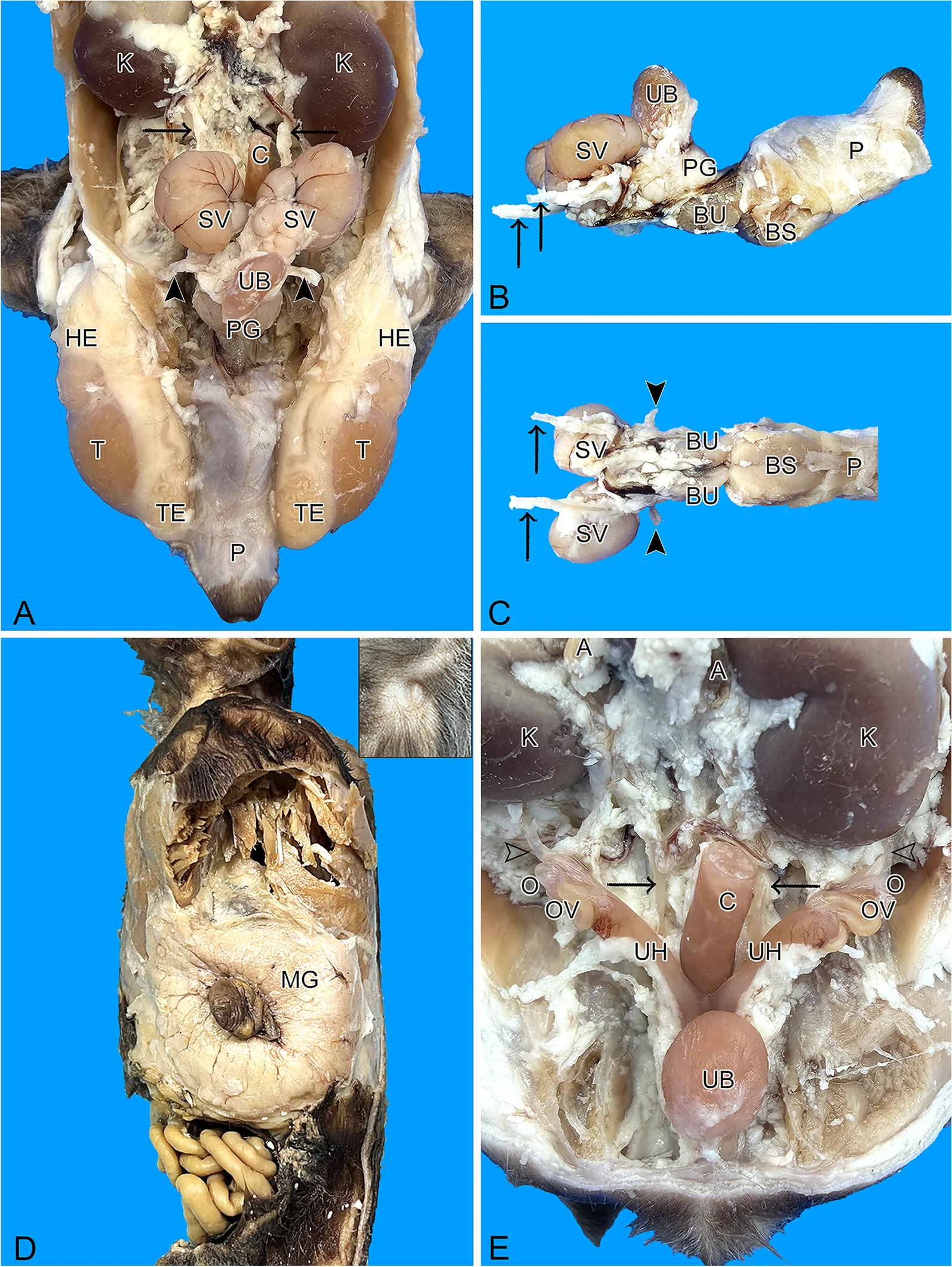
**Gross features of the male and female reproductive tract in Egyptian rousettes (*Rousettus aegyptiacus***). **A** Ventral aspect of the adult male reproductive tract in situ. Note that the overlying gastrointestinal, splenic, and hepatic tissue has been removed here and in panel **E**. **B** Right lateral aspect of a portion of the adult male reproductive tract ex situ. **C** Dorsal aspect of the adult male reproductive tract ex situ. Note the tip of the penis is not included in the image. **D** Left lateral view of mammary gland with central nipple in an adult female. Note the left limb and wing membrane have been removed. Inset: Right lateral view of vestigial nipple in an adult male. **E** Ventral aspect of the adult female reproductive tract in situ. Note that the ERBs shown in all but panel **D** are from a managed-care research colony and therefore have more subcutaneous and visceral adipose than would be present in a free-ranging ERB. The open arrowhead indicates the suspensory ligament, the arrow the ureter, and the arrowhead the vas deferens. A, adrenal gland; BS,  bulbospongiosus muscle; BU, bulbourethral gland; C = colon; HE, head of the epididymis; K, kidney; MG, mammary gland; O, ovary; OV, oviduct; P, penis; PG, prostate gland; SV, seminal vesicle; T, teste; TE, tail of the epididymis; UB, urinary bladder; UH, uterine horn.

**Figure 10:**
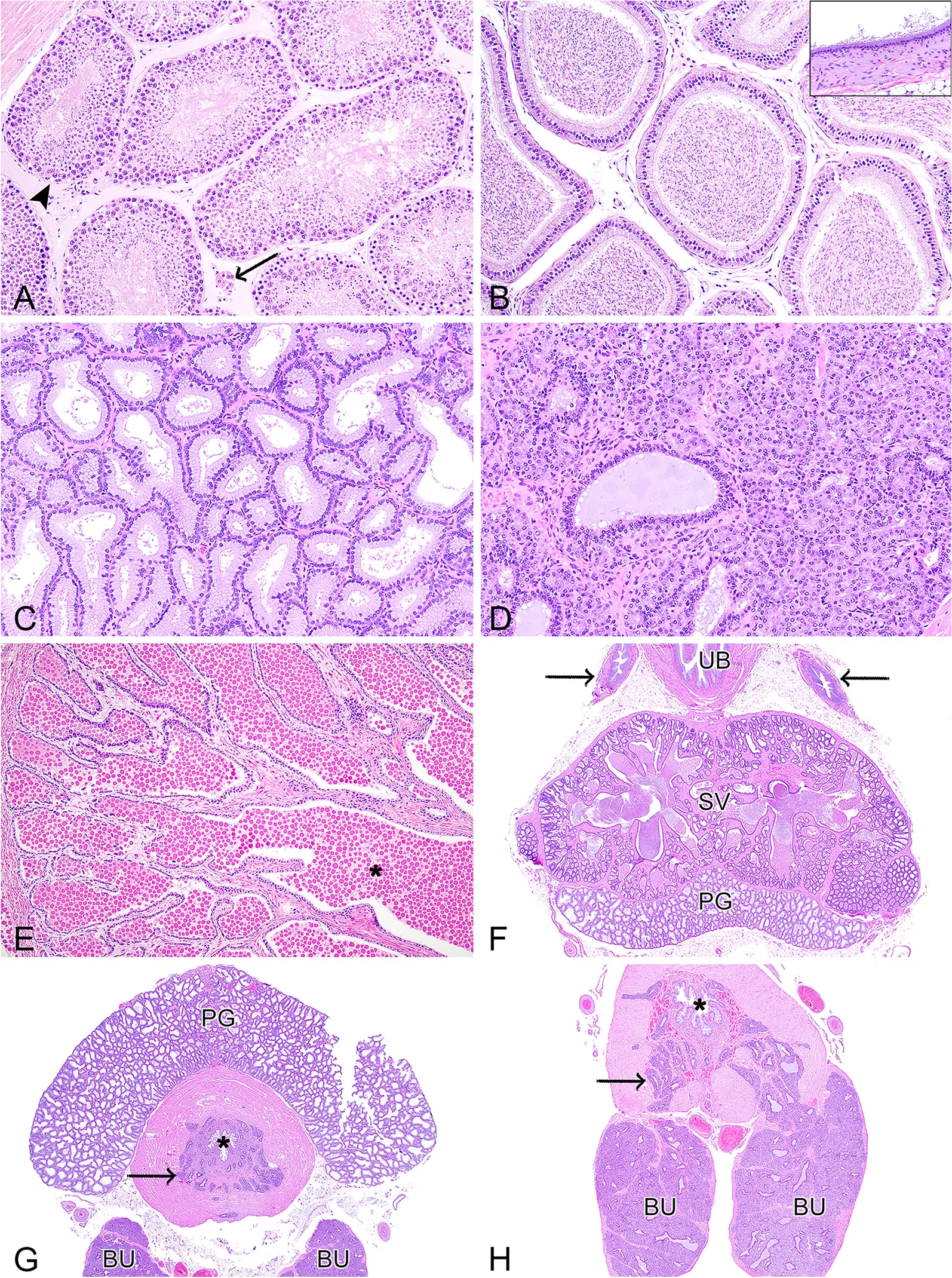
**Histologic features of the male reproductive tract in Egyptian rousettes (*Rousettus aegyptiacus***). **A** Seminiferous tubules in the teste of an adult male. The germinal epithelium contains Sertoli cells and developing spermatocytes; testosterone-producing Leydig cells (arrow) are present within the interstitial space, and myoid cells (arrowhead) surround the tubules. ×20, H&E. **B** Epididymal duct of an adult male, lined by tall pseudostratified columnar epithelium with apical stereocilia and intraluminal spermatozoa. ×20, H&E. Inset: Vas deferens in an adult male, taken at the level of the body of the epididymis. ×40, H&E. **C** Prostate gland in an adult male. Tubules composed of tall columnar epithelium are supported by a fibromuscular stroma. ×10, H&E. **D** Bulbourethral/Cowper's gland in an adult male, composed of numerous tubules lined by simple columnar epithelium. ×20, H&E. **E** Seminal vesicle with highly proteinaceous intraluminal seminal material (asterisk) in an adult male. ×10, H&E. **F** Cross-section of the caudal aspect of the seminal vesicle (SV) at the level of the prostate gland (PG) and urinary bladder (UB) in an adult male. The arrow indicates the ureter. ×2, H&E. **G** Cross-section of the prostate gland (PG) at the level of the bulbourethral glands (BU), caudal to the section in panel **F**. Note the intramuscular course of the ducts of the bulbourethral glands (arrow) to access the urethral lumen (asterisk). The tissue fracture within the prostate gland is a sectioning artifact. ×2, H&E. **H** Cross-section of the bulbourethral glands (BU) and urethra, caudal to the section in panel **G**. The arrow indicates the bulbourethral gland ducts and the asterisk the urethral lumen. ×2, H&E.

Testes are abdominal in juvenile males and scrotal in adults (Figures 9A, 10A, B). Aside from expected differences in organ size, no consistent age-associated histologic differences were identified in this dataset; however, detailed assessment of reproductive maturation (e.g., spermatogenic staging or follicular development) was beyond the scope of this study. In ERBs, studies of reproductive seasonality note that testicular recrudescence and associated size increases occur seasonally and are tightly linked to mating activity in adults, but do not provide detailed morphologic comparisons across age classes within a population [118]. Accessory reproductive structures include a prostate gland incompletely surrounding the urethra (Figures 9A, B, 10C, F, G), Cowper's glands/bulbourethral glands (Figures 9B, C, 10D, G, H), seminal vesicles (Figures 9A-C, 10E, F), and ampullary glands [119, 120]. Although an ampullary gland within the vas deferens has been reported in this species, we were unable to confidently identify this structure on either gross or histologic examination. The penis comprises corpus spongiosum, prominent corpora cavernosa, a relatively large and well-developed glans penis compared with other chiropterans, and a small os penis residing fully within the glans, covered by thin, retractile prepuce slightly extending beyond the glans tip when flaccid (Figures 8H, 9A, B) [119]. An accessory corpora cavernosa is absent [121]. Male ERBs have nonfunctional and underdeveloped nipples bilaterally on their lateral thorax (Figure 9D, inset).

### Female reproductive system

**Figure 11:**
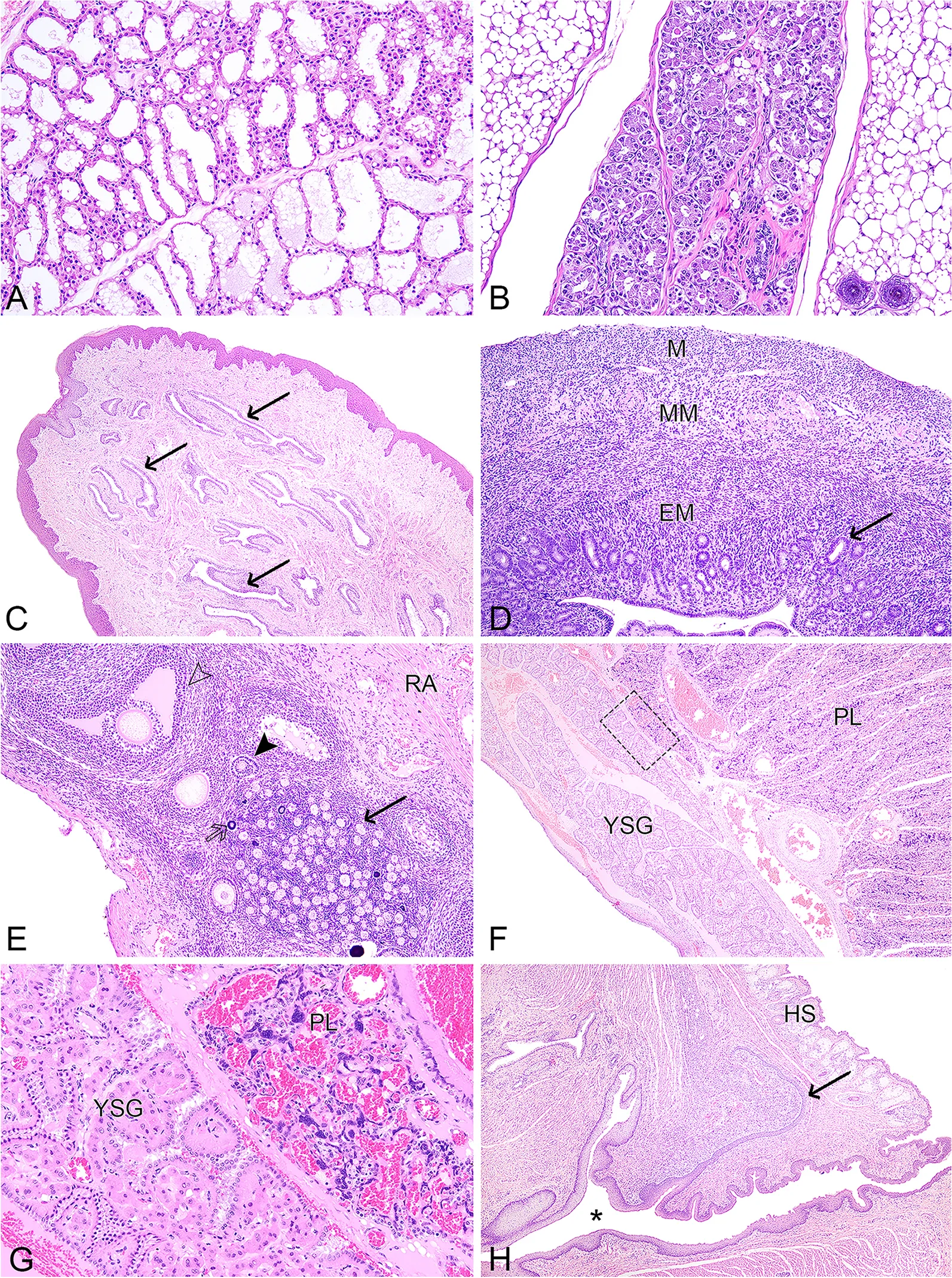
**Histologic features of the female reproductive tract in Egyptian rousettes (*Rousettus aegyptiacus***). **A** Active mammary tissue composed of numerous tubuloacinar secretory units in an adult female. ×20, H&E. **B** Inactive mammary tissue in a juvenile female. ×20, H&E. **C** Longitudinal section of nipple with numerous ducts (arrows) in an adult female. ×4, H&E. **D** Uterine cross-section from a non-gravid adult female with endometrial glands (arrow) within the endometrium (EM), vascular mesometrium (MM), and myometrium (M). ×10, H&E. **E** Ovarian cortex with numerous primordial follicles (arrow), fewer primary follicles (arrowhead), and one secondary follicle (open arrowhead), with adjacent rete arteriosum (RA) in an adult female. Follicular mineralization (open arrow) can be seen as part of normal atresia [130]. ×10, H&E. **F** Near-term placenta with placental labyrinth (PL) and yolk-sac gland (YSG) in an adult female. Tissue inside the dashed rectangle is shown in panel **G**. ×4, H&E. **G** Near-term placenta with placental labyrinth (PL) and yolk-sac gland (YSG) in an adult female. ×20, H&E. **H** Longitudinal section of vaginal vestibule (asterisk) and clitoris (arrow) in an adult female. HS, haired skin. ×4 H&E.

Females have concentric mammary tissue bilaterally on lateral thoracic aspects with central nipples (Figures 9D, 11A-C). The uterus (Figures 9E, 11D) is duplex and symmetrical; two uterine horns are externally caudally united, but their lumina open at the vagina through separate cervical canals [122–124]. *R. leschenaultii* bats have been reported to exhibit human-like menstrual cycles [125]. Reproductive asymmetry is common in Pteropodidae [123, 126]. During pregnancy, a single corpus luteum occurs on the same reproductive tract side as the developing embryo and persists until subsequent ovulation; the contralateral ovary contains primary and secondary follicles (Figure 11E) [123, 126]. Delayed embryonic implantation has been reported in at least three chiropteran families [127] and has been anecdotally observed in ERB managed care colonies (J. Towner, personal communication). No histologic findings in the present study provided direct evidence to support or refute the occurrence of delayed embryonic implantation in this species. *Rousettus* spp. bats exhibit discoid, labyrinthine, and hemochorial placentation with intrasyncytial lamina and solid glandular yolk sacs at term (Figure 11F, G) [128, 129]. A broad, flattened clitoris is present within the genital tubercle (Figure 11H) [124].

## Lymphoid

**Figure 12:**
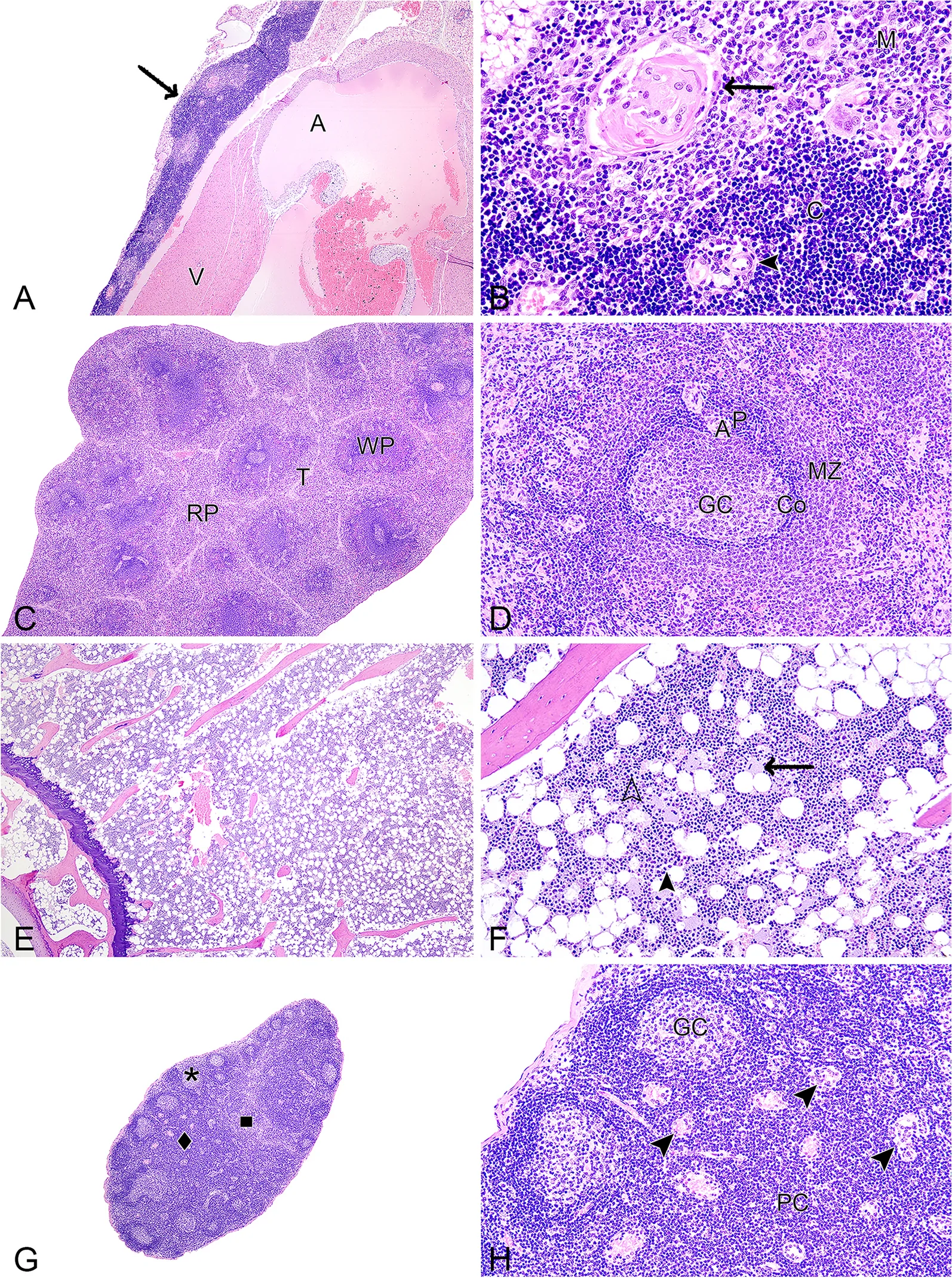
**Histologic features of thymus, spleen, bone marrow, and lymph node in Egyptian rousettes (*Rousettus aegyptiacus***). **A** Left lobe of the thymus (arrow) wrapping around the left atrium (A) and left ventricle (V) in a juvenile male. ×4, H&E. **B** Hassall's corpuscle (arrow) in the thymic medulla (M) of a juvenile male. The arrowhead indicates the capillaries. C, cortex. ×40, H&E. **C** Spleen with white pulp containing numerous lymphoid follicles (WP), red pulp (RP), and smooth muscle trabeculae (T) in a juvenile male. ×4, H&E. **D** Adjacent to numerous central arteries (A) surrounded by a periarteriolar lymphoid sheath (P) is a splenic lymphoid follicle with a central pale germinal center (GC) and an outer deeply basophilic corona/mantle zone (Co), further surrounded by a marginal zone (MZ), which delineates the boundary between white pulp and red pulp. Juvenile male, ×20, H&E. **E** Bone marrow at the medullary cavity of the femoral head of a juvenile female. ×2, H&E. **F** Erythroid (open arrowhead) and lymphoid (arrowhead) precursors admixed with megakaryocytes (arrow) and adipose at the femoral head of a juvenile female. ×20, H&E. **G** Lymph node with numerous cortical secondary follicles (asterisk) overlying paracortical regions (diamond) and medullary sinus (square) in an adult female. ×4, H&E. **H** B-cell-rich secondary follicles with germinal centers (GC) in the cortex overly an indistinct T-cell-rich paracortex (PC), with numerous high endothelial venules (arrowheads) in an adult female. ×20, H&E.

Previous gross and histologic analyses have demonstrated that bats have similar primary and secondary lymphoid organs as other mammals, including thymus (Figure 12A, B), spleen (Figure 12C, D), bone marrow (Figure 12E, F), and lymph nodes (Figures 3A, B, 12G, H), plus widely dispersed immune cells [131–135]. Gross anatomical lymph node characterization in ERBs has been documented, with symmetrical anatomical locations and variation in numbers as follows: superficial cervical (1-2), facial (2-4), internal jugular (1-3), posterior cervical (1-4), brachial (1-3), axillary (1-4), inguinal (2-6), popliteal (2-3), gluteal (2-5), iliac (1-4), renal (1-3), and cranial mesenteric (1-5) [136, 137]. Pregnant ERBs have been reported to demonstrate more numerous and enlarged lymph nodes than nonpregnant females or males, with typically larger left-sided lymph nodes [136]. This may reflect immunologic remodeling of existing lymphoid tissues, including enlargement and altered cellular composition of uterine-draining and peripheral lymph nodes, rather than the formation of new lymph nodes [138].

Splenic structure resembles humans and rodents, featuring thin capsules and variably sized collagenous and smooth muscle trabeculae [139]. *R. leschenaultii* bats have few erythroid and myeloid cells and no megakaryocytes in red pulp, with prominent white pulp marginal zones [139], findings that are consistent with those observed in the present study. Splenic size may decrease in response to stress [109].

## Discussion

This study provides a comprehensive histologic atlas of the Egyptian rousette, integrating original morphologic observations from wild-caught animals with previously published literature into a single, standardized reference framework. Although this species is central to investigations of zoonotic disease ecology, particularly as the natural reservoir of Marburg virus, baseline tissue architecture has remained inconsistently documented, limiting interpretation of experimental infection studies and spontaneous disease processes. By providing normal histologic features across major organ systems, this work establishes a practical framework to support comparative, diagnostic, and experimental pathology and to aid investigators and pathologists in distinguishing normal tissue architecture from disease-associated change in Egyptian rousettes examined in field investigations, experimental studies, and comparative mammalian research.

Routine hematoxylin and eosin histology limits evaluation to overall tissue architecture and broad cellular patterns. Extension of these observations using immunophenotyping, in situ hybridization, and emerging spatial transcriptomic approaches will be valuable for defining cellular composition and functional microenvironments, particularly within lymphoid, mucosal, and neurovascular tissues. Such methods may prove especially informative for mapping immune cell distributions and viral localization, thereby linking morphologic organization with host defense mechanisms. Incorporation of digital pathology platforms and advanced imaging modalities may further enhance accessibility of reference materials and permit quantitative or three-dimensional assessment of tissue architecture.

Interpretation of normal versus pathologic morphology would also benefit from expanded evaluation across developmental stages, reproductive states, and environmental contexts. Comparative analyses incorporating neonatal, adult, and geriatric cohorts, as well as animals maintained under differing nutritional or managed-care conditions, could clarify physiologic variability associated with maturation, reproduction, or stress. Longitudinal sampling strategies and controlled experimental studies may further elucidate dynamic changes in immune organization and organ remodeling over time.

Several limitations of this study warrant consideration. Specimens were subjected to prolonged fixation in 10% neutral buffered formalin for approximately 15 years, followed by transfer to 70% ethanol at an unknown time point. This preservation history likely introduced artifacts affecting staining characteristics, nuclear-cytoplasmic contrast, tissue dimensions, and lipid preservation, particularly given that carcasses were fixed intact. These factors may have limited assessment of overall morphologic quality in certain tissues.

The study population was also biased toward juvenile animals (49 juveniles versus 20 adults), with no representation of neonatal or geriatric individuals. This distribution reflects the opportunistic nature of specimen acquisition from wild-caught populations rather than targeted demographic sampling. Consequently, structures subject to age-related remodeling, including thymus, bone marrow, lymphoid organs, and hepatic tissue, may not fully represent changes occurring during early development or senescence. Future studies incorporating age-stratified cohorts will be necessary to more comprehensively characterize developmental and maturational variation in this species.

Additionally, tissues were collected during a single seasonal period, limiting evaluation of potential seasonal influences such as reproductive cycling, endocrine variation, or fluctuating pathogen exposure. Although the number of animals examined represents a substantial cohort for wildlife histologic studies, it may not capture the full range of histologic variability across the species- broad geographic distribution. Future investigations incorporating prospectively collected specimens with broader demographic and geographic representation will further refine understanding of histologic variability within this species.

Despite these constraints, the present atlas provides a robust baseline for interpreting tissue morphology in healthy, free-ranging Egyptian rousettes. By enabling accurate distinction between normal architecture and disease-associated change, this resource supports future studies addressing host-pathogen interactions, comparative mammalian biology, and mechanisms underlying pathogen tolerance in natural reservoir species. As additional specimens and analytical approaches become available, continued refinement of this reference framework will further enhance its utility for both wildlife pathology and biomedical research.

## Data Availability

No datasets were generated or analyzed during the current study.
